# Nursing students’ learning strategies for e-learning during the Covid-19 pandemic in Iran: a qualitative study

**DOI:** 10.1186/s12909-023-04270-9

**Published:** 2023-05-08

**Authors:** Nesa Cheraghbeigi, Shahram Molavynejad, Dariush Rokhafroz, Nasrin Elahi, Eisa Rezaei

**Affiliations:** 1 Student Research Committee, School of Nursing and Midwifery, Ahvaz Jundishapur University of Medical Sciences, Ahvaz, Iran; 2 Nursing Care Research Center in Chronic Diseases, School of Nursing and Midwifery, Ahvaz Jundishapur University of Medical Sciences, Ahvaz, Iran; 3 Department of Educational Technology in Medical Sciences, Smart University of Medical Sciences, Tehran, Iran

**Keywords:** Covid-19 pandemic, e-learning, Learning strategies, Nursing students

## Abstract

**Background:**

In response to the emergency brought about by the Covid-19 pandemic, many universities around the world had to change their teaching methods from in-person classes to e-learning. The purpose of this study was to identify the learning strategies of nursing students in e-learning during the pandemic.

**Methods:**

This study had a qualitative design and used content analysis approach to collect and analyze the data. Sixteen semi-structured interviews were conducted with 12 Iranian undergraduate nursing students who were selected using purposive sampling method.

**Results:**

Most nursing students in this study generally used two different strategies for e-learning, namely self-centered learning strategies and collaborative learning strategies. Some students, on the other hand, adopted a passive approach in which they did not take any effective action to contribute to their learning.

**Conclusion:**

In e-learning during the pandemic, students adopted different learning strategies. Therefore, designing teaching strategies tailored to the students’ strategies can promote their learning and academic achievement. Also, knowledge of these strategies helps policy makers and nursing educators to take necessary measures in order to optimize and facilitate student learning in an e-learning environment.

## Background

With the sudden emergence of the Covid-19 pandemic, all sectors of society including education experienced widespread disruptions [1]. Nursing education, as a key component of higher education, was no exception, and with the unexpected emergence of Covid-19, nursing educators were urged to make quick decisions on how nursing education should be continued, and all solutions they came up with were nothing but to quickly replace in-person education with electronic education [2].

In Iran, before the outbreak of the pandemic, the method of teaching medical sciences, including nursing education, was through face-to-face education, and electronic education had no specific place in the strategic plans of universities. Nevertheless, in response to the closure of universities and in order to avoid a debilitating pause in the education process of students, the Ministry of Health, Treatment and Medical Education called for the rapid presentation of electronic education in universities of medical sciences, including nursing schools, and in this way, electronic education quickly became compulsory in all universities of the country [2].

The sudden and unexpected transition from face-to-face to electronic education undoubtedly made students face various challenges and obstacles, and several studies have identified these challenges. Moradi et al. (2022) in their qualitative study, for example, reported the following as the main challenges that nursing students and professors went through in this respect: lack of necessary infrastructure for e-learning, insufficient facilities and equipment, financial problems, professors’ unpreparedness to use e-learning, reluctance of students and professors to use e-learning, and the professors’ insufficient adherence to e-learning [3]. In addition, increased workload, anxiety, being away from the clinical environment, insufficient communication and interactions between students and professors, problems related to educational content, superficial learning, and inability to develop critical thinking have been cited as other problems of students in e-learning during the pandemic [2, 4]. Therefore, in the face of these challenges, the students were urged to adopt certain strategies to facilitate their learning [5]. Although many studies have been conducted on learning strategies in face-to-face education [6] and in conventional online education, few have dealt with the extent to which the use of these strategies in mandatory electronic education during the pandemic period is beneficial to students. This is particularly important with respect to nursing students owing to the unique nature of their field of study (which involves both theoretical and clinical aspects) [5].

Moreover, the sudden implementation of e-learning during the Covid-19 pandemic created a unique context that was definitely different from what has been reported in previous studies on e-learning in terms of the scale and participation of participants. That is, in studies conducted before the pandemic, online courses were mainly offered on a voluntary basis, or they were only mandatory for a certain number of students [7]. On the other hand, most of the studies that have been conducted on learning styles of students in e-learning are quantitative in approach and use self-report questionnaires such as the Kolb Learning Style Inventory, which divides individuals into predetermined categories for data collection [8, 9]. By contrast, qualitative studies allow researchers to gain a deeper understanding of the phenomenon under investigation, and such studies can provide valuable insights into the special conditions caused by the Covid-19 pandemic. In fact, studies using qualitative approaches enable professors to take effective measures in order to facilitate their students’ learning in distance education and to help them learn with more independence and motivation. Therefore, the present qualitative study aimed to identify learning strategies used by undergraduate nursing students in online education offered in response to the Covid-19 pandemic in Iran.

## Methods

### Setting

The present study was conducted at Ahvaz University of Medical Sciences in Iran in 2022. During the COVID-19 pandemic, a national offline learning management system, known as NAVID, was used in this university. Furthermore, two more computer applications used for distance education, namely “Adobe Connect” and “Skyroom”, were used as online education methods to support education offered through NAVID. Adobe Connect and Skyroom are two popular and practical software for holding conferences, meetings, or synchronous training classes online. They allow the participants to share their picture, voice, and desktop.

### Design

In this study, inductive content analysis method was used to identify nursing students’ learning strategies for e-learning during the Covid-19 pandemic. Due to its exploratory nature, content analysis approach enables researchers to gain a deeper understanding of the phenomena under study and discover their complexities [10].

### Participants

The statistical population of this study included undergraduate nursing students of Ahvaz Jundishapur University of Medical Sciences, Ahvaz, Iran. Twelve undergraduate nursing students were selected using purposive method from among eligible students. The inclusion criteria were: having at least two academic semesters of e-learning experience and willingness to participate in the study. Maximum variation sampling technique was used to capture a diverse range of views and experiences. Therefore, sampling was done from both sexes and from second- to fourth-year students. The research sample included 3 s-year students, 5 third-year students, and 4 fourth-year students. Also, the majority (n = 9) of the students included in the study were female. The age of the participants ranged from 22 to 24 years.

### Data collection

Semi-structured interviews were used to collect the data. Compared to quantitative methods of data collection, interviews provide deeper insights into the phenomenon under study. Interview questions included, but were not limited to, the following:

1- Could you please explain your experience of e-learning?

2- Could you please explain how you attended e-learning nursing courses?

3- How did you learn in this semester or the previous semesters when your courses were offered electronically?

In addition to open questions, probing questions were also used in order to delve into the learning experiences of the participants. Also, at the end of each interview, the participants were given the opportunity to add more points to their statements if they wished. The interviews were conducted by the first author (NC), who is a PhD student in nursing. She had passed advanced courses on research methods in her PhD program and had conducted several interviews in partial fulfillment of those courses. In addition, she conducted the first interview in this study under the supervision of the supervisor (the corresponding author of the present study). After her qualification to conduct qualitative interviews was verified by the supervisor, she proceeded to perform the subsequent interviews. The data collection process continued until data saturation. In cases of any ambiguity, a follow-up interview was also set up. In general, in our study, after the transcripts of the interviews were read several times during and after the data analysis process, the following cases of ambiguity were detected: incomprehensible statements, comprehensible statements with inadequate information, and statements with multiple interpretations. After these cases of ambiguity were identified, they were resolved through consultation with the research team, which involved asking specific questions from the same interviewee or the next one for confirmation of the correct interpretation of the data. A total of 16 face-to-face interviews with 12 undergraduate nursing students were conducted. Nine students were interviewed once, one student was interviewed three times, and two students were interviewed twice. The reason why some of the interviewees were interviewed more than once was due to the fact that after analyzing the interview transcripts of some of the participants, we realized that the obtained data was inadequate, and in some cases, was not completely clear to us. Therefore, since each of these cases of ambiguity could have a profound contribution to our results and since we had access to all participants, we re-interviewed some the students. The interviews were conducted individually in one of the classrooms of the School of Nursing and Midwifery in a quiet and peaceful atmosphere. The mean length of the conducted interviews was 65 min. The interviews were recorded using a digital audio recorder. The content of each interview was transcribed verbatim immediately after the interview.

### Data analysis

The process of data analysis was done simultaneously with data collection. Inductive content analysis as explained by Graneheim and Lundma was used for data analysis which involved the following five stages: 1- Verbatim transcription of each interview immediately after its completion, 2- Reading the entire transcribed text in order to get a general understanding of the content of the interview, 3- Determination of basic codes and semantic units, 4- Classification of primary codes into broader categories based on their similarities, 5- Determining the main theme of the categories [11].

In the present study, after the completion of each interview, the entire recorded audio file was listened to carefully. After that, the content of the interview was transcribed verbatim in Microsoft Word software. Then, to immerse in the data and to extract the main themes, the interview transcript was read several times. MAXQDA 2020 was used for data management. The first author identified the semantic units by reading the interview text line by line. Semantic units were converted into primary codes. Afterwards, the codes were compared based on similarities and differences and were categorized into themes and sub-themes. Any disagreements regarding codes, themes and sub-themes were resolved by discussion sessions held by the research team. Finally, after the themes and sub-themes were refined, they were specified in the text.

### Rigor

The four criteria of Guba and Lincoln, namely credibility, confirmability, dependability, and transferability were used to achieve validity and reliability of the data [12]. The first author’s long engagement with the data ensured credibility. That is, during the data analysis process, which lasted six months from May 12, 2022 to November 15, 2022, the researcher repeatedly read the transcript of the interviews. In addition, an expert supervisor supervised the data analysis process and any disagreement was resolved by discussion and agreement. In addition to peer-check, member-check was also used to achieve credibility. To this aim, a summary of the findings was provided to the participants after analyzing each interview, and they were asked to check whether the concepts obtained reflected their experiences or ideas. If any additional comments or experiences were added by the participants, they were included in the analysis. Also, maximum variation sampling technique was used which involved choosing both male and female students studying in different academic years. In order to ensure confirmability and facilitate auditability, a complete description of the research process, including data collection and analysis methods, was presented. Dependability was confirmed by inviting an external audit to check the similarities and differences between the researchers’ and participants’ understanding of the topics. Through complete and accurate description of the participants, the time and place of data collection and the sampling method, the researchers tried to achieve transferability of the data.

## Results

Our data analysis showed that students’ learning strategies for e-learning during the pandemic fall into three main themes of “self-centered learning strategies”, “collaborative learning strategies” and “remaining passive” (Table 1). Using direct quotes from the participants, the themes and their sub-themes are presented below.

**Table 1:** Students’ learning strategies in e-learning

Theme	Sub-theme	Code
Self-centered learning strategies	Exploration	Searching the Internet
		Asking peers
		Asking teachers
	Using evidence	Studying reference textbooks
		Studying relevant articles
	Using educational media and tools	Attending educational webinars
		Using educational systems and software
		Using educational Telegram channels
	Using audio-visual resources	Watching educational videos
		Listening to narrated PowerPoint slides
	Dedicating a place for studying	Dedicating a quiet and comfortable place for studying
		Dedicating a particular place for studying
	Summarizing and note-taking	Classification and summarization of content
		Note-taking
	Reflection on the materials taught	Reflection on the narrated PowerPoint slides
		Reflection on educational videos
	Skimming	Skimming the content in the first round of studying
		Trying to understand the main idea
	Individual role playing	Playing the role of a teacher
		Teaching to hypothetical students
Collaborative learning strategies	Collaborative note-taking and pamphlet writing	Forming groups for note-taking and pamphlet writing
		Division of responsibilities in note-taking and pamphlet writing
		Uploading the group pamphlet
	Peer-to-peer teaching and learning	Forming a group of peers
		Division of teaching materials between group members
		Teaching the materials to one another
Remaining passive	Giving up and not taking action	Giving up
		Not taking action

### Theme 1: self-centered learning strategies

This theme includes the following 9 sub-themes: “Exploration”, “Using evidence”, “Using educational tools and media”, “Using audio-visual resources”, “Dedicating a particular space for studying”, “Summarizing and taking notes”, “Reflection on the content taught”, “Skimming” and “Individual role playing”.

#### Exploration

Many students stated that they used this strategy when they were faced with some professors’ inadequate teaching and when some topics raised questions or were ambiguous for them. Exploration manifested itself in a variety of behaviors that include “searching the Internet”, “asking the teacher” and “asking peers”. However, the students’ first choice to solve their learning problems was to search the Internet, which was followed by questions from peers and questions from teachers. The following quotes shed more light on how and why these solutions were adopted by the students.

*“I used to search and download educational videos available on Google. For example, when the teacher taught CPR, I did not learn it very well and didn’t know where exactly I should place these leads. That’s why I searched the Internet and found a related video.” (Participant 7, 7th semester female student)*

*“If I happened to have a problem, I would first search the Internet, and if I couldn’t find the answer, I would ask one of the classmates who knew the answer.” (Participant 9, 8th semester female student)*

#### Using evidence

Based on the experiences of our participants, using evidence involved reading reference textbooks and reading relevant scientific articles. The students used this strategy when the topics were inadequately presented by the professor, or when they did not understand the topics well despite the professor’s good teaching.

*“For example, I had the professor’s pamphlet and the content file, but I felt that the teaching materials were not complete, so I used to supplement those materials from different books to help my learning.” (Participant 8, 6th semester female student)*

*“For some of things that I had problem with such as some points in anatomy, I would look up the answer in books. Or in physiology, for example, I again studied the heart physiology from scratch so that I could understand the different axes. Although the teacher had taught very well, I myself felt that I did not understand, so I read the book again. Or I used the materials and articles that were available on the Internet.” (Participant 3, 4th semester female student)*

#### Using educational tools and media

According to the experiences of our participants, this strategy included participation in educational webinars, using educational systems and software (*Full Code Medical Simulation*, *Labster*, and *Faradars*), and using Telegram educational channels. Some students stated that in order to better learn the topics in which they felt weak, they participated in educational webinars, which often cost them money:

*“Yeah, these webinars are very good. I myself attended a webinar on pharmacology. Of course, I paid a fee to participate, and then the topics were taught very nicely; for example, beta blockers or calcium blockers; these were taught in a nice way using diagrams along with audio files explaining the topics very well. I myself participated in these. For example, I was weak in the pharmacology course, so I participated in these and it was very good.” (Participant 1, 4th semester female student)*

In addition, since practical/clinical topics were not presented to students efficiently in electronic education, some students, especially those who had already been familiar with the basics of virtual education, used the strategy of using simulator software and systems to learn these topics.

*“Yeah, these apps helped me a lot, because you can go step by step. I had never been to hospital, but when I was working with Full Code, although it was in English, I understood that as a nurse, I am allowed to do some examinations. I mean, I used it to learn physical examination. In addition to Full Code, there is another system called Labster, which is comprehensively designed so that you can wear gloves and a gown, or plug the microscope in, all in that simulation space. So much attention has been paid to the details! This app was one of the things that I used for the laboratory courses.” (Participant 5, 5th semester female student)*

#### Using audio-visual resources

Another strategy adopted by the interviewed students was using audio-visual educational resources. This included watching educational videos and listening to narrated PowerPoint slides. This strategy was mostly used by students who had a visual-auditory learning style.

*“Because I myself learn better through videos, I prefer watching videos. For example, I found a video clip about CPR. I saw that clip and it was very good.” (Participant 12, 6th semester male student)*

*“I have a habit of having someone explain things to me, and I listen to learn. What I used to do was listen to the teacher’s voice. When I was idle, I used to listen to the teacher’s voice. In my mind, when I heard the teacher’s voice it looked as if the lesson had been repeated for me. Lessons that were repeated were very good.” (Participant 8, 6th semester female student)*

#### Summarizing and taking notes

Another self-centered learning strategy which the students participating in the present study used, especially those with a reading/writing learning style, was summarizing and note-taking. The students stated that the flexibility of e-learning facilitated the use of this strategy. Taking notes and summarizing helped these students to review the educational material faster when studying for final exams.

*“Well, I’m used to writing in order to learn. In e-learning, I had more time to categorize the materials I was supposed to learn. I made a table for myself. The night before the exam, for example, I could easily distinguish between different diseases or the symptoms of different diseases by reading them on a table. It took me much less time.” (Participant 4, 6th semester female student)*

#### Dedicating a particular place for studying

Some students stated that they had dedicated a particular place for studying to facilitate their learning. Such a place, according to the participants, had to be quiet and comfortable.

*“I used to choose a place to study that was comfortable and without noise. Besides, it had to be a particular place so that my brain would know that it was time to study. Well, this made me learn better and faster.” (Participant 2, 4th semester female student)*

#### Reflection

Reflection on the educational materials was another self-centered learning strategy adopted by some students to learn the topics more deeply.

*“After I listened to the professor’s narrated PowerPoint [slide] or watched an educational video, I would think about those contents and make an analysis for myself of that PowerPoint [slide] or video” (Participant 6, 5th semester female student)*

#### Skimming

Some students believed that using the skimming strategy led to better learning of the topics. This strategy helped them understand the main idea of the educational material and learn the topics more quickly the next time they studied the same content.

*“Another thing I do is, for example, to read the entire article quickly and then come back to the beginning. Now that I have read it once, I have a general idea about it, and then I read it again in more detail. This makes me learn better.” (Participant 8, 6th semester female student)*

#### Individual role playing

Playing the role of a teacher was another self-centered learning strategy used by some students. These students stated that for a better and deeper understanding of the course material, they imagined themselves to be one of the professors and then taught the topics to hypothetical students.

*“One of the methods I used, in addition to summarizing the teacher’s voice, was to play the role of the teacher for myself by giving a lecture. I mean, I imagined myself as a teacher and put myself in shoes of Professor X or Y, and I would explain what he or she said to me, as if I had students and I was the professor and I was teaching the lesson to my students. This way, my efficiency would be much higher.” (Participant 7, 7th semester female student)*

### Theme 2: collaborative learning strategies

This theme has two sub-themes, namely “peer-to-peer teaching and learning” and “collaborative note-taking and pamphlet writing”.

#### Peer-to-peer teaching and learning

The results of our study showed that some students, especially those who had the desire to learn socially, formed groups of peers to facilitate their learning, and after dividing the topics among each team member and achieving mastery over the specified topics, they would teach each other. The use of this strategy led to reduced student workload and better learning owing to the division of responsibilities and the comprehensibility of peer explanations.

*“Sometimes it was even like each of us would, for example, teach one chapter to the others. For example, if we had five chapters and there were five of us in a group, then each one of us would take one chapter. Well, this way, the work was divided among everyone. On the other hand, because each of the chapters we were responsible for was read very well, when we taught it, it was comprehensible to others.” (Participant 6, 7th semester female student)*

#### Collaborative note-taking and pamphlet writing

Collaborative note-taking and pamphlet writing was another collaborative strategy employed by the students. The final product of this strategy was a complete and concise educational pamphlet, which significantly reduced the students’ workload and made it easier for them to study for the final exams.

*“We had a note-taking and pamphlet writing group, and we had a class representative for note-taking and pamphlet writing. This representative would divide the class into smaller groups, and each group was responsible for a certain topic, which had to be written down based on that teacher’s voice and PowerPoint [slides], and then finally the representative would combine all these pamphlets and put them in the pamphlet group. This note-taking and pamphlet writing was very good because the contents were complete, and it was much easier to read at the night before the exam.” (Participant 9, 8th semester female student)*

## Theme 3: remaining passive

This theme involves two sub-themes, namely “Giving up” and “Not taking action”. The results of our study showed that some students felt lost in the face of the challenges of e-learning and did not take any effective action to help their learning.

*“I didn’t do anything special. The amount of material was so large that one would get confused. I would just read some topics the night before the exam only to pass the exam. It was really bad. I would keep praying that the in-person education would resume as soon as possible.” (Participant 11, 6th semester male student)*

## Discussion

The aim of the current study was to identify the learning strategies employed by undergraduate nursing students in online education provided in response to the Covid-19 pandemic in Iran. According to the results of our study, these students used self-centered and collaborative strategies to facilitate their e-learning during the pandemic. Some students, on the other hand, chose to remain passive. In the following, these findings are discussed in more details and in separate sections.

### Self-directed learning strategies

The results of our study showed that students used various self-centered strategies to facilitate their e-learning. In fact, due to the teacher’s physical absence in the e-learning environment, students have more independence in the learning process, and in order to be able to learn and succeed in this type of education, it is necessary to have self-regulation learning skills [13]. In fact, studies related to e-learning have also found a positive relationship between self-directed learning strategies, academic success, and the e-learning acceptance [14, 15]. Wesselborg et al. (2020) concluded that promoting health care students’ self-centered learning during the COVID-19 pandemic helps them not only to use their learning opportunities systematically but also to be able to plan their learning activities in a goal-oriented way [16]. In our study, some students stated that a small number of their professors provided them with valuable guidance in this regard; of course, after the students themselves had directly requested them to do so. Although learning in an online environment requires students to be highly motivated and equipped with self-regulated learning skills, some students may lack such prerequisites. Therefore, it is imperative that such students be supported by their teachers in order to overcome the challenges they face and to ensure their success in online education [17].

The present study identified several self-directed learning strategies, two of which worth special attention and are discussed below. In addition, Table 2 provides additional information on how the existing literature supports the learning strategies discovered in this study. One of the self-centered strategies used by some students in our study was exploration, which is consistent with the results of Kee et al. (2020) [18]. Similar to our study, another study also showed that students are more willing to get response of their queries from their peers, and a smaller percentage of students talk to professors [19]. One of the reasons why more students prefer the strategies of searching the Internet and asking peers over the strategy of asking the professor can be related to the shorter time of receiving answers by the first two strategies [20]. Nevertheless, some students in our study admitted that a small number of their teachers provided their private numbers so that the students could call them in case of any ambiguity. A more focused analysis of the data revealed that this strategy was adopted by some professors in order to facilitate the learning process of students to compensate for the insufficient communication and interactions in e-learning during the pandemic. However, there are no institutional rules in this regard, which may raise concern about privacy issues. Therefore, it is worth mentioning that without any clear institutional rules and guidelines, it is very difficult for both students and teachers to set boundaries between professional teaching and learning and their private lives. As Khan et al. (2022) reported on remote education experiences during the pandemic, faculty members were under immense pressure to give their private number to students [21]. Therefore, in order to protect one’s privacy in online education environments, it is very essential that educational institutions develop certain guidelines and policies in this regard.

**Table 2:** How the existing literature supports the learning strategies discovered in the present study

Learning strategies identified in the present study		support by Existing literature
**Self-centered learning strategies**	Exploration (Searching the Internet, asking peers, asking teachers)	-greater willingness of students to ask peers and search the Internet than the strategy of asking teachers [19]. - shorter time to get answers by searching the internet and asking peers [19, 20].
	Using evidence (Studying reference textbooks, studying relevant articles)	-Reference textbooks are rich educational resources [20]. -Students are able to obtain reliable knowledge and information through reference textbooks [20]. - Students can expand their knowledge through searching authentic educational articles [33].
	Using educational media and tools (Attending educational webinars, using educational systems and software, using educational Telegram channels)	-Using webinars in combination with virtual simulators increase the involvement of nursing students in e-learning process and also their clinical reasoning [34]. - webinar and virtual simulators can simulate face to face education for students [35]. - Webinars allow professors to be in contact with their students simultaneously and directly, through audiovisual communication, and to provide them with immediate feedback [35]. - the possibility of forming discussion groups and educational channels with a very large number of members in Telegram, facilitate students’ access to educational resources and thereby help them learn better [36].
	Using audio-visual resources (Watching educational videos, listening to narrated PowerPoint slides)	- approximately 75% of learning occurs through seeing and 13% through hearing [23]. - audio-visual teaching materials can help learners to reduce their learning time and retrieve information more efficiently [2]. - Educational videos and narrated PowerPoint slides enable students to learn anywhere and as many times as they wish [2].
	Dedicating a place for studying	- students are satisfied with e-learning platforms when they use them in an environment that promotes e-learning [37]. - not learning in a structured and controlled environment when using e-learning, students need to structure their physical learning environment so that they are less distracted and can concentrate better [38].
	Summarizing and note-taking	- The flexibility of e-learning methods can facilitate the learning of students who prefer to learn by writing and taking notes [20]. - summarizing helps students to retrieve essential information from among a multitude of educational materials in a relatively short period of time [39].
	Reflection on the materials taught	- reflection and learning skills are important factors in the students’ acceptance of e-learning [40]. - Reflection plays a very important role in nursing students’ learning, and turning them into critical and autonomous nurses [41].
	Skimming	- using skimming as a reading technique by nursing students is associated with a better understanding of the material [42]. - skimming helps students to predict the purpose and the main idea of the text they are studying [43].
	Individual role playing	- Role-playing is a fun and stimulating strategy for students [44]. - Role-playing allows students to step outside of their role as students and is associated with a better understanding of subjects due to the active role of the student in the learning process [44].
**Collaborative learning strategies**	Collaborative note-taking and pamphlet writing	- The students’ use of collaborative methods allows them to share their ideas with each other, divide responsibilities and devise plans. therefore, manage the workload more effectively [45].
	Peer-to-peer teaching and learning	- In peer-to-peer education, students will be more at ease to ask questions and then get feedback [28]. - Peer explanations are more comprehensible and it is easier for students to acquire knowledge which is more durable [28].

As another self-centered learning strategy, the students participating in this study used audio-visual educational resources, which included watching educational videos and listening to narrated PowerPoint slides. Hampton et al. (2016) reported that educational videos and PowerPoint slides narrated by nursing students who participated in an e-learning program were the most engaging and effective learning methods [22]. Results of research investigating the contribution of the human senses to learning indicate that approximately 75% of learning occurs through seeing and 13% through hearing [23]. In addition, the use of audio-visual teaching materials can help learners to reduce their learning time and retrieve information more efficiently [24]. Educational videos and narrated PowerPoint slides enable students to learn anywhere and as many times as they wish by providing a learning environment with no time and space limitations, with the possibility to repeat the content [2].

### Collaborative learning strategies

Apart from strategies in which the learner played the central role, the results of our study showed that some students used certain collaborative learning strategies, which is in line with the results of Dewi et al. (2021) who showed that collaborative learning strategies in e-learning during COVID- 19 pandemic were positively associated with motivation, length of learning, and student comprehension [25]. Furthermore, the results of a systematic review revealed that collaborative learning in digital learning contexts helps enhancing nursing students’ knowledge, satisfaction, competence, and problem solving skills [26].

One of the collaborative learning strategies in our study that is particularly worthy of discussion was teaching to and learning from peers. In agreement with our study, the results of previous studies confirm that peer-to-peer teaching in e-learning improves student learning [27, 28]. In peer-to-peer education, students will be more at ease to ask questions and then get feedback, and since the peer explanations are more comprehensible, it is easier for students to acquire knowledge which is more durable [28]. In our study, peer-learning was self-initiated, and the group members were chosen by the members themselves. Therefore, some students might not have had the opportunity to benefit from such groups. Although the many benefits of informal peer learning are hardly disputable, this type of learning should be made more explicit and embedded into higher education courses so that more students can benefit from its potential advantages. Of course, relying solely on peer learning is neither possible nor appropriate. However, careful and gradual use of peer-to-peer education can greatly impact learning process in a positive way [29].

### Remaining passive

One of the noteworthy results of our study was that some students assumed a passive role in facing the challenges that e-learning had created for them during the pandemic, and they did not take any special action to promote their learning. An in-depth examination of the data revealed that the inability of some students to work with the necessary technologies, inadequate facilities and equipment, poor financial status, living in less privileged areas, inadequate self-directed learning skills, and the desire to learn through the teacher in the classroom led these students to give up and adopt a passive approach. Similar to our findings, the results of a study by Yeung et al. (2022) in Hong Kong also indicated that some students felt lost in online education during the pandemic and surrendered to its challenges [5]. Also, Smith et al. (2014) in their study reported a large number of students who did not participate at all in asynchronous or live e-learning environments [30]. Therefore, it is very imperative that professors and policy makers take appropriate measures to support this group of students who do not have the necessary skills and facilities to manage their learning in online education.

### Gender differences in learning preferences

The majority of the participants in our study were female, which may have skewed the results of the study. In fact, a large body of literature has examined gender differences in learning. It is beyond the scope of our study to provide a comprehensive overview in this regard. However, some points are briefly mentioned here. In general, females and males are different in a variety of areas and learning is only one of them. Nevertheless, it is believed that females prefer detailed processing in learning. Therefore, when the materials are taught, they try to establish a personal connection with them in order to learn better. On the other hand, men are more inclined to rational evaluation while learning. Furthermore, it has been reported that interaction with peers and self-confidence in learning are more important for female students than for males. In other words, unlike female students who are more social and tend to be function-oriented, male students seem to be more achievement-centered [31, 32].

### Strengths and limitations

To the best of our knowledge, the present study is the first to identify the learning strategies of Iranian nursing students in the context of e-learning during the Covid-19 pandemic. Nevertheless, in our study, we identified the learning strategies used by a particular group of Iranian nursing students in e-learning. Therefore, the findings of this study cannot be generalized to other settings and populations. This is because students may have different demographic characteristics and online learning experiences in different settings. Furthermore, the majority of the participants were female, and the number of students from each academic year was relatively small, which are other limitations in our study that may have skewed the results.

### Suggestions for future studies

More studies are recommended to be conducted in other countries in order to shed more light on the nature of students’ learning strategies for e-learning. Also, results of comparisons between strategies adopted by students from different countries can be used to help the students become independent e-learners. Moreover, since the majority of the participants in the present study were females, it is recommended that future studies recruit an equal number of participants from both sexes.

## Conclusion

Using a content analysis approach, our study attempted to identify certain strategies adopted by Iranian nursing students in e-learning during the difficult conditions caused by COVID- 19 pandemic. According to our results, nursing students adopted different self-centered and collaborative learning strategies. Nevertheless, it is important to mention that the results of the study indicated some students surrendered to the challenges of e-learning and had a passive approach. Therefore, knowledge of these strategies helps policy makers and nursing educators to take necessary measures in order to optimize and facilitate student learning in an e-learning environment. Also, designing teaching strategies tailored to the students’ strategies can promote their learning and academic achievement.

## Data Availability

Access to the data obtained from this research is available upon reasonable request from the corresponding author.
